# Developing the educational needs of Telehealthcare support staff at SCQF/QCF level 6

**Published:** 2012-06-15

**Authors:** Audrey Cund, Donna Henderson, Doreen Watson, Donna Fleming, John Honeyman, Fiona Fraser, Polly Wright

**Affiliations:** University of Strathclyde, UK; Joint Improvement Team, UK; Joint Improvement Team, UK; Edinburgh City Council, UK; Fife Council, UK; NHS education for Scotland, UK; Middlesborough Council, UK

**Keywords:** education, telehealthcare staff development, competency framework

## Abstract

**Background:**

The use, monitoring and care of individuals through the use of technology have played a key role in the delivery of health and social care services for decades (Wooton et al. 2006). Contemporary changes in how health and social care is provided in Scotland have focussed on using Telehealthcare as a medium to improve access, efficiency and equity of services across the country for a range of service users and the management of a range of conditions (JIT 2011). The Scottish Government Joint Improvement Team (JIT) and the Scottish Centre for Telehealth (SCT) published an Education and Training Strategy for Telehealthcare in Scotland in March 2010. The Strategy acknowledged that the provision of education and training to support unqualified staff working in telehealthcare service delivery was limited to locally developed, non-accredited training delivered in-house by telehealthcare service providers. It identified that although accredited frameworks do exist in Higher Education at SCQF/QCF 9, 10 and 11, the context, content and academic level was not appropriate for support staff. To meet the education and training needs of unqualified staff working in telehealthcare, the Strategy Action Plan included a workstream to develop a Competency Framework for Telehealthcare Support Staff. A subsequent workstream within the Action Plan focussed on the design and development of an academic award based on the competencies identified within the new Framework. This involved setting up a Qualification Design Team (QDT) made up of telehealthcare and education service providers and specialists. The Qualification Design Team worked with the Scottish Qualifications Authority (SQA) to design and accredit a Professional Development Award (PDA) in Telehealthcare at SCQF/QCF level 6. This paper outlines the design and development of the Professional Development Award.

**Project objectives:**

1. To design a Professional Development Award in Telehealthcare to meet the educational needs of unqualified support staff working in telehealthcare service delivery.
2. To achieve National accreditation of a Professional Development Award in Telehealthcare at SCQF/QCF level 6.

**Proposed next stage objectives:**

1. To evaluate the telehealthcare support staff experience of undertaking the Professional Development Award.
2. To evaluate the modes of delivery and geographical contexts in which the Professional Development Award is delivered.
3. To describe the impact the educational experience has on the candidates personal and professional development.

**Proposed design/method:**

A mixed method research design will be used to evaluate the candidate’s experiences of undertaking the Professional Development Award in Telehealthcare. Structured interviews and surveys will be used to gather information to analyse the candidate’s experience. A random sample of candidates will be drawn from all SQA approved sites to deliver the Professional Development Award.

**Findings and points of interest:**

It is anticipated that a positive outcome will be achieved from the project evaluation as this award was driven and led by Telehealthcare staff working in the field. Further awards and specific modules are likely to emerge from this work to underpin advancements in career pathways and to address innovations and developments in telehealthcare.

